# Articular Eminence Inclination, Height, and Condyle Morphology on Cone Beam Computed Tomography

**DOI:** 10.1155/2014/761714

**Published:** 2014-02-13

**Authors:** Dilhan İlgüy, Mehmet İlgüy, Erdoğan Fişekçioğlu, Semanur Dölekoğlu, Nilüfer Ersan

**Affiliations:** Department of Dentomaxillofacial Radiology, Faculty of Dentistry, Yeditepe University, Bağdat Caddesi No. 238, Göztepe Kadıköy, 34728 Istanbul, Turkey

## Abstract

*Aim*. The aim of the present study was to examine the relationship between articular eminence inclination, height, and thickness of the roof of the glenoid fossa (RGF) according to age and gender and to assess condyle morphology including incidental findings of osseous characteristics associated with osteoarthritis (OA) of the temporomandibular joint (TMJ) using cone beam computed tomography (CBCT). *Materials and Methods*. CBCT images of 105 patients were evaluated retrospectively. For articular eminence inclination and height, axial views on which the condylar processes were seen with their widest mediolateral extent being used as a reference view for secondary reconstruction. Condyle morphology was categorized both in the sagittal and coronal plane. *Results*. The mean values of eminence inclination and height of males were higher than those of females (*P* < 0.05). There were significant differences in the RGF thickness in relation to sagittal condyle morphology. Among the group of OA, the mean value of the RGF thickness for “OA-osteophyte” group was the highest (1.59 mm), whereas the lowest RGF values were seen in the “OA-flattening.” *Conclusion*. The sagittal osteoarthritic changes may have an effect on RGF thickness by mechanical stimulation and changed stress distribution. Gender has a significant effect on eminence height (Eh) and inclination.

## 1. Introduction

The glenoid fossa, located at the inferior aspect of the squamous part of temporal bone, is composed of the glenoid fossa and articular eminence of the temporal bone. It is sometimes described as the temporal component of the temporomandibular joint (TMJ). The articular eminence forms the anterior limit of the glenoid fossa and is convex in shape [1]. The articular eminence is a part of the temporal bone on which the condylar process slides during mandibular movements. The inclination of articular eminence varies among people and it dictates the path of condylar movement as well as the degree of rotation of the disc over the condyle [2, 3]. Fossa depth varies, and the development of the articular eminence relies on functional stimulus from the condyle [1]. The thickness of the roof of the glenoid fossa (RGF) of the TMJ was assessed in previous studies related to gender, age, and condyle morphology [4–6]. Tsuruta et al. [7] suggested that compensative bone formation in the RGF might help to withstand the increased stress in the TMJ accompanying condylar bone change, especially erosion. The changes of osteoarthritis seen in the TMJ, such as erosion, articular surface remodelling and flattening, and osteophytes, are identical to those seen in other affected joints. As is typical of this condition, the changes seen on imaging do not always correlate with symptoms. Many patients may be pain-free despite advanced osteoarthritis and the only complaint is of joint noises or grating [8].

In previous studies, cone beam computed tomography (CBCT) was used to detect the inclination of articular eminence and condyle morphology [4, 9]. CBCT can be recommended as a dose-sparing technique compared with standard medical computed tomography scans for dentomaxillofacial imaging [10]. Studies have suggested that CBCT provides accurate and reliable linear measurements for reconstruction and imaging of dental and maxillofacial structures [11, 12]. A review of the published literature revealed no studies on the relationship between the measurements of articular eminence and condyle morphology using CBCT. The aim of the present study was to examine the relationship between articular eminence inclination, height, and thickness of the roof of the glenoid fossa (RGF) according to age and gender and assess condyle morphology including incidental findings of osseous characteristics associated with osteoarthritis (OA) of the TMJ using CBCT.

## 2. Materials and Methods

The study received approval from the Ethics Committee in Research of Yeditepe University (Protocol no. 334/2013). The retrospective study group was planned according to Sample Size Estimation Simple Random Sampling and consisted of CBCT scans of 105 asymptomatic patients. Forty of the patients were males and 65 were females and the images were obtained from the archive of the Dentomaxillofacial Radiology Department of the Faculty. Patient ages ranged from 18 to 80 years. Patients with a fracture or pathology in the region of the articular eminence, which interferes with performing the measurement on the region, were not included in the study. Digital images were taken using an ILUMA CBCT scanner (Imtec Corporation, Oberursel, Germany) with an amorphous silicon flat-panel image detector and a cylindrical volume of reconstruction of up to 19 × 24 cm. Images were taken at 120 kVp, 3.8 mA, and a voxel size of 0.2 mm, with an exposure time of 40 seconds. 3D reconstructions were created by reformatting the axial CBCT scans on a local workstation using ILUMA dental imaging software (Imtec Corporation, Oberursel, Germany). A written informed consent form was also signed, which is routinely obtained from each patient prior to imaging in our faculty. Before measurements were made, the orientation of the images was determined for each patient. The Frankfort plane (a line passing horizontally from the superior border of external auditory meatus to the inferior border of the orbital rim) was held parallel to the horizontal plane on the lateral view.

A specialist in dentomaxillofacial radiology assessed the images in a darkened quiet room with dual monitors (HP LP2475W, resolution 1920 × 1200). The contrast and brightness of the images were adjusted using the image processing tool in the software to ensure optimal visualization. Three measurements were made on the monitor and the mean value was calculated for statistical analysis. For the prevention of a possible technical error, the measurements of the images were repeated by the same observer for a second time after 2 weeks.

**Figure 1:**
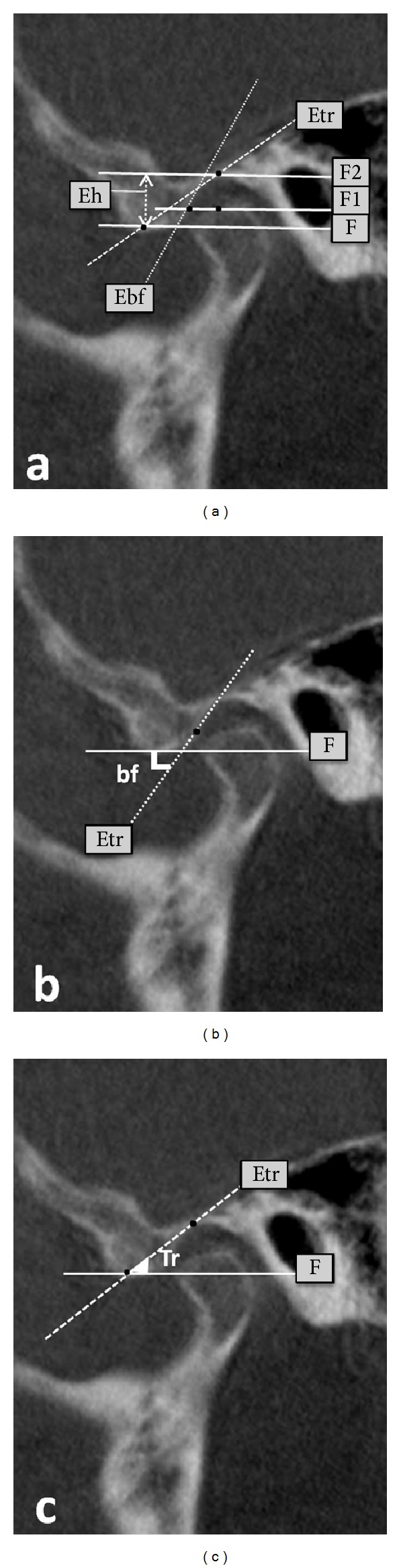
(a) The points and planes used in this study, (b) the best-fit line method, and (c) the top-roof line method. F: Frankfort horizontal, F1: the parallel line to the F passing through the highest point of the condylar process, F2: the parallel line to the F passing through the highest point of the fossa, Ebf plane: the best-fit plane of the articular eminence inclination connecting the point, at which the F1 line cut the eminence posterior surface, Etr plane: the plane passing through the highest point of the condylar process and the highest point of the condylar process, and Eh: Eminence height.

For articular eminence inclination and height, one of the axial views on which the condylar processes were seen with their widest mediolateral extent was used as a reference view for secondary reconstruction. The points and planes used in this study were as follows: Ce: the point at which the F1 line cut the eminence posterior surface; Cu: the highest point of the condylar process; Po: porion (the highest point of auditory meatus); R: the highest point of the fossa; T: the lowest point of the articular eminence; Ebf plane: the best-fit plane of the articular eminence inclination connecting the Ce; Etr plane: the plane passing through the points Cu and R; F: Frankfort horizontal; F1: the parallel line to the F passing through the point Cu; and F2: the parallel line to the F passing through the point R. The eminence inclination was measured by two methods (Figure 1(a)). The first was the best-fit line method that was the angle between Ebf and Frankfort horizontal (Figure 1(b)); the second was the top-roof line method that was the angle between Etr and Frankfort horizontal (Figure 1(c)) [9].

The eminence height (Eh) was measured by the perpendicular distance between the lowest point of the articular eminence and the highest point of the fossa (Figure 1(a)). For the detection of the thinnest RGF, the central equivalent region of RGF was identified among the coronal slices on the monitor and a distance measurement tool was used to measure the thickness (Figure 1) [4]. The measurements were performed on the central sagittal slice.

**Figure 2:**
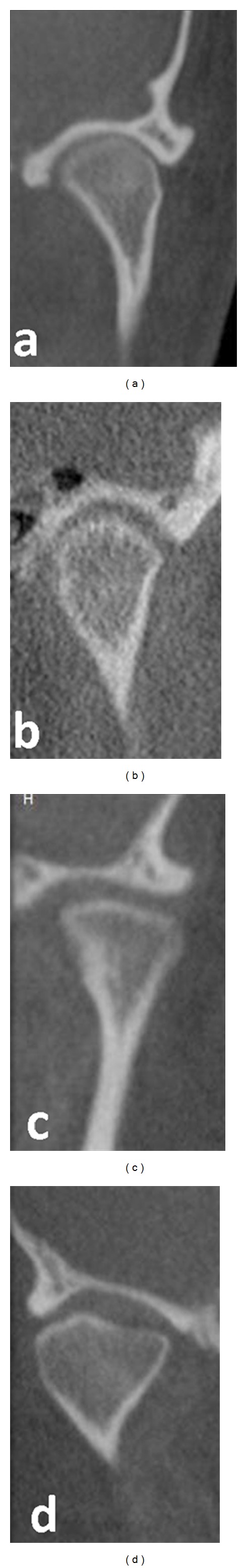
The coronal condyle morphology: (a) convex, (b) round, (c) flat, and (d) angled and other.

**Figure 3:**
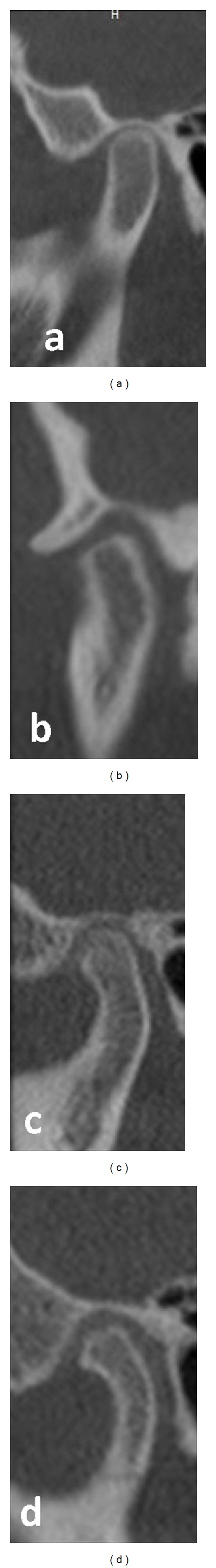
The sagittal condyle morphology: (a) without osteoarthritis (OA), (b) flattening, (c) erosion, and (d) osteophyte.

Condyle morphology was categorized both in the sagittal and coronal plane [4]. The coronal plane was set parallel to the long axis of the condyle and the sagittal plane was set perpendicular to the coronal one. The condyles were classified as “convex,” “round,” “flat,” “angled”, and “other” in the coronal plane described by Ejima et al. [4] (Figures 2(a), 2(b), 2(c), and 2(d)). They were also grouped as without osteoarthritis (OA) or with OA (flattening/osteophyte/erosion) in the sagittal plane (Figures 3(a), 3(b), 3(c), and 3(d)). All data were recorded on both sides. Consequently, 210 sites were analyzed altogether. Also, the numbers of remaining teeth on both anterior and posterior regions were recorded for each patient. The patients who had prosthetic rehabilitation were excluded from the study.

Data were analyzed using the Statistical Package for Social Sciences (SPSS, IBM, New York, USA) 15.0 software for Windows. Kappa value was used to determine the internal consistency. During the evaluation of the study data, along with the descriptive statistical methods, parameters with normal distribution for the comparison of quantitative data were evaluated using one-way ANOVA and Tukey HSD test. Student's *t*-test was also used for parameters with normal distribution. Kruskal-Wallis test was used for comparing samples that are independent. The specific sample pairs for significant differences were analyzed with Mann-Whitney *U* test. The strength of relationship between two variables was measured with Spearman rho correlation coefficient. Significance was accepted at *P* < 0.05 level.

## 3. Results

The mean age of the study group was 47.47 ± 17.78 years. Using Kappa value, the internal consistency of the interratings of the observer for best-fit line, top-roof line, RGF thickness, and eminence height was found to be 0.94, 0.92, 0.90, and 0.95, respectively (*P* < 0.05).

The metric parameters of the articular eminence and RGF thickness according to gender are shown in Table 1. The mean values of eminence inclination and height of males were higher than those of females (*P* < 0.05). No statistically significant difference was found between RGF thickness and gender (*P* > 0.05).

The study group was divided into age groups and there was a statistically significant difference between age groups and measurements of eminence inclination and RGF thickness (Table 2, *P* < 0.05). According to the Tukey HSD test, the mean values of inclination in the 30–39-year age group (54.22 ± 6.02 and 44.73 ± 7.12) were found to be statistically highest among age groups (*P* < 0.05). The 40–49-year age group had the highest value of the RGF thickness (*P* < 0.05). No statistically significant difference was found between RGF thickness and age groups (*P* > 0.05).

In terms of coronal condyle morphology, no relation was found with the measurements of articular eminence and RGF thickness (*P* > 0.05). There were significant differences in the RGF thickness in relation to sagittal condyle morphology (Table 3). Among the group of OA, The mean value of the RGF thickness for “OA-osteophyte” group was the highest (1.59 mm), whereas the lowest RGF values were seen in the “OA-flattening” (1.21 mm, Mann-Whitney *U* test).

Spearman rho correlation coefficient revealed that there was no significant correlation between the number of remaining teeth and the measurements of articular eminence on both anterior and posterior regions (*P* > 0.05).

## 4. Discussion

The articular eminence is located in front of the glenoid fossa and its posterior surface slope varies among people [1]. It is subjected to functional load arising from chewing forces, and the morphological shape of it may be affected by these loads [13].

In previous studies, different methods were used to measure the inclination of the posterior slope of the articular eminence. Studies performed with no slices may not show a true measurement of eminence inclination [14]. The view of the eminence in the central slice is the steepest part of the eminence. For true measurement of eminence inclination, it is the best view of eminence inclination [9, 15] and, in the present study, the central sagittal slice of the condylar process was used for measurements.

The articular eminence may predispose to disc displacement. The shape of the articular eminence is related to the development of TMJ disc displacement [16].

Conventional techniques are inadequate for TMJ imaging because of the anatomical complexity of this region. Previous studies have stated that CBCT provides accurate and reliable linear measurements for reconstruction and imaging of dental and maxillofacial structures and it has shown superior results for imaging the TMJ [11, 17, 18]. In the present study, CBCT was used for the measurements of articular eminence and condyle morphology.

The articular eminence inclination completes about 90–94% of its growth by the age of 20 years [19]. Previous studies reported that morphological changes may occur in the eminence structure with advanced age and this results in the flattening of the eminence in the long term [14, 16]. In contrast, in some studies, no correlation was found between advanced age and eminence anatomy for either eminence height or inclination [9, 20]. Our results are consistent with these studies because, in older age groups, there was no significant difference related to measurements of articular eminence.

Previous publications reported a significant correlation between the thickness of the RGF and sagittal condyle morphological characterization [4, 7, 21]. Honda et al. [22] stated that mechanical stimulation may cause an increase in bone thickness in the glenoid fossa because of an incomplete shock absorption function resulting from perforation of the disc or altered retrodiscal connective tissue. In the present study, a significant correlation was found between the thickness of the RGF and sagittal condyle morphology; OA-osteophyte group had the thickest RGF.

Some studies found a difference in eminence inclination according to gender [4, 20, 23]. In the present study, eminence inclination and height values of males were higher compared to females (Table 1). In contrast to previous studies, no relation was found between RGF thickness and gender in the present study [4–6]. The results of the present study (male 1.26 mm, female 1.24 mm) appear to be lower than those reported in these studies. These differences may be attributed to the sample sizes used in these studies.

Factors influencing stress distribution in the condyle region were investigated in earlier studies and revealed that morphological changes in the condyle head could alter the stress distribution in the region of RGF. It was also previously reported that mechanical stimulation might cause an increase in bone thickness in the glenoid fossa [22]. According to our results, a significant correlation was found between RGF thickness and sagittal condyle morphology. These results are consistent with these studies, which showed a difference between RGF thickness and sagittal condyle morphology [4, 7, 21]. Ishimaru et al. [24] concluded that, in sheep, malocclusion had no effect on the normal TMJ. Ejima et al. [4] found no significant correlation between the remaining teeth and RGF thickness. In the present study, the remaining teeth were investigated on two regions of mouth. According to our results, it was observed that remaining teeth on both anterior and posterior site had no influence on the RGF thickness. These data are consistent with the results of the previous study [4], which demonstrated no association between RGF thickness and remaining teeth.

In conclusion, the sagittal osteoarthritic changes may have an effect on RGF thickness by mechanical stimulation and changed stress distribution. Articular eminence inclination has no relation with age. Gender has a significant effect on eminence height and inclination. This anatomical data may be helpful to understand the articular eminence anatomy. Additional studies on a larger number of cases are needed due to the variation, which might be present between different populations which may affect the articular eminence measurements.

## Figures and Tables

**Table 1:** The articular eminence and RGF thickness measurements according to gender.

	Male	Female	*P*
	Mean ± SD	Mean ± SD	
Best-fit line (°)	49.66 ± 6.88	47.58 ± 6.75	0.033*
Top-roof line (°)	40.19 ± 6.58	37.99 ± 6.00	0.014*
RGF thickness (mm)	1.26 ± 0.42	1.24 ± 0.38	***0.717***
Height (mm)	7.33 ± 1.26	6.69 ± 1.16	0.001**

Student's *t*-test; **P < 0.01; *P < 0.05.

**Table 2:** The articular eminence and RGF thickness measurements according to age groups.

	Age groups					*P*
	<30	30–39	40–49	50–59	≥60	
	Mean ± SD	Mean ± SD	Mean ± SD	Mean ± SD	Mean ± SD	
Best-fit line (°)	46.02 ± 7.65	54.22 ± 6.02	46.45 ± 6.06	46.91 ± 5.16	48.59 ± 6.42	0.001**
Top-roof line (°)	38.17 ± 7.07	44.73 ± 7.12	36.71 ± 5.0	37.17 ± 4.33	38.08 ± 5.18	0.001**
RGF thickness (mm)	1.20 ± 0.32	1.37 ± 0.48	1.49 ± 0.47	1.16 ± 0.37	1.18 ± 0.33	0.001**
Art eminence height (mm)	6.64 ± 1.47	7.19 ± 1.09	6.66 ± 1.35	6.91 ± 1.05	7.11 ± 1.19	***0.169***

One-way ANOVA test; **P < 0.01.

**Table 3:** The mean values of the articular eminence and roof of the glenoid fossa thickness according to sagittal condyle morphology.

	Sagittal condyle morphology				*P*
	Without OA	With OA			
		Flattened	Osteophyte	Erosion	
Best-fit line (°)	48.50 ± 7.0	48.06 ± 6.90	50.13 ± 5.31	48.44 ± 7.25	***0.785***
Top-roof line (°)	39.15 ± 6.98	38.6 ± 5.94	38.41 ± 3.01	38.04 ± 6.72	***0.963***
RGF thickness (mm)	1.24 ± 0.37	1.21 ± 0.39	1.59 ± 0.28	1.48 ± 0.78	0.014*
Height (mm)	6.74 ± 1.10	7.05 ± 1.33	7.07 ± 0.91	7.44 ± 1.82	***0.361***

Kruskal-Wallis test; *P < 0.05.
